# Supplementary material for: Factors associated with virological non-suppression among HIV-positive children receiving antiretroviral therapy at the Joint Clinical Research Centre in Lubowa, Kampala Uganda
Source: PLoS One. 2021 Jan 27;16(1):e0246140. doi: 10.1371/journal.pone.0246140 (PMC7840004; doi:10.1371/journal.pone.0246140)
Supplement: S1 File — (DOCX) [file pone.0246140.s002.docx]

**DATA ABSTRACTION FORM TO ASSESS FACTORS ASSOCIATED WITH VIROLOGICAL NON-SUPPRESSION AMONG CHILDREN LIVING WITH HIV RECEIVING ART AT THE PEDIATRIC HIV/AIDS CLINIC AT JCRC.**

**DATE OF DATA COLLECTION: ………………………………………………………………….**

**NAME OF THE DATA COLLECTOR: …………………………………………………………….**

Child’s identification Number ………………………………………………….

When did the child start ART?

DATE………. MONTH………...YEAR……………….

What was the child’s age in complete years at initiation of ART?

……………………………………………………………………………………………

What is the Child’s gender? Use a tick

Female ……………………………………………………………………

Male ...………………………………………………………………...

Is the child on TB-treatment or received TB- treatment in the past one year? Use a tick.

‘YES’ …………………………………………………………………………………….

‘NO……………………………………………………………………………………….

What was the child’s WHO clinical stage at the beginning of ART? Tick the correct option.

Stage I …………………………………………………………………………………….

Stage II …………………………………………………………………………………….

Stage III ……………………………………………………………………………………

Stage IV ……………………………………………………………………………………

What was the child’s initial/first Viral load (copies/mL)?

……………………………………………………………………………………..

What was the first viral load measurement (copies/ml) at or after 6 months of treatment

………………………………………………………………………………………

…………………………………………………………………………………………….

Which ART treatment line is the child receiving?

First-line ………………………………………………………………………………

Second-line …………………………………………………………………………….

Third –line ………………………………………………………………………………

ii). What was the reason for switching to second- line or to third-line?

…………………………………………………………………………………………………..

…………………………………………………………………………………………………….

What ART –regimen is the child currently receiving? Tick the correct option

Efavirenz –based ……………………………………………………………….

Nevirapine – based ………………………………………………………………...

Lopinavir/ritonavir – based ……………………………………………………

What is the child’s adherence level in the first 6 months of ART?

……………………………………………………………………….

Has the child registered any ART-related side effects in the past 2 years?

YES………………………………………………………………………………………...

IF yes, please mention the side-effects ………………………………………………

……………………………………………………………………………………………….

**- END**
